# Plac8-ERK pathway modulation of monocyte function in sepsis

**DOI:** 10.1038/s41420-024-02012-4

**Published:** 2024-07-03

**Authors:** Teng Zhang, Jing-nan Fu, Gui-bing Chen, Xiu Zhang

**Affiliations:** 1 Department of General Surgery, Tianjin Medical University General Hospital, Tianjin, 300000 China; 2 Department of Minimally Invasive Surgery, Characteristics Medical Center of Chinese People Armed Police Force, Tianjin, China; 3 Department of General Surgery, Clinical Medical College and The First Affiliated Hospital of Chengdu Medical College, Chengdu, China; 4 Department of Emergency, Beijing Tsinghua Changgung Hospital, Beijing, 102218 China

**Keywords:** Medical research, Paediatric research

## Abstract

Sepsis, a life-threatening condition caused by infection, is characterized by the dysregulation of immune responses and activation of monocytes. Plac8, a protein, has been implicated in various inflammatory conditions. This study aimed to investigate the effect of Plac8 upregulation on monocyte proliferation and activation in sepsis patients. Peripheral blood samples were collected from healthy individuals and sepsis patients. Monocytes were stimulated with lipopolysaccharide (LPS) to create an in vitro sepsis model, while a murine sepsis model was established using cecal ligation and puncture (CLP). The levels of monocyte markers, proliferation index (PI), and pro-inflammatory cytokines were assessed using flow cytometry and qPCR, respectively. Plac8 and phosphorylated ERK protein levels were determined by western blot, and TNF-α, IL-6, and IL-10 levels were quantified using ELISA. The CCK-8 assay was used to evaluate PBMC proliferation and activation. The results showed that Plac8 was highly expressed in sepsis models, promoting the survival, proliferation, and activation of monocytes. Plac8 upregulation activated the ERK pathway, leading to increased phosphorylation of ERK protein and elevated levels of CD14, CD16, TNF-α, IL-6, Plac8, and IL-10. In sepsis mice, Plac8 overexpression similarly activated the ERK pathway and promoted the survival, proliferation, and activation of monocytes. In conclusion, the upregulation of Plac8 enhances the activation of the ERK pathway and promotes monocyte proliferation and activation in sepsis patients.

## Introduction

Sepsis is a life-threatening disease caused by an uncontrolled host response to infection, and it remains a major cause of death and severe illness worldwide, imposing a significant burden on global healthcare systems [1–3]. Despite advances in antimicrobial therapy and intensive care techniques, the incidence of sepsis continues to rise, attributed to the aging population and the increasing prevalence of antibiotic-resistant pathogens [2, 4, 5]. The complexity of sepsis arises from its heterogeneity, with diverse clinical manifestations ranging from systemic inflammatory response to severe septic shock and multiple organ dysfunction syndrome [6, 7]. Dysregulation of immune responses plays a central role in the pathophysiology of sepsis, and peripheral blood mononuclear cells (PBMCs) play a key role in this process [8–10]. Cells, including monocytes, lymphocytes, and dendritic cells, play important roles in balancing pro-inflammatory and anti-inflammatory responses [11, 12]. However, due to a limited understanding of the regulatory mechanisms governing PBMC function during the acute and recovery phases of sepsis, the clinical management of sepsis has become complex, highlighting the need for innovative research in this field.

Monocytes, a crucial subset of PBMCs, play a dual role in sepsis by directing innate immune responses through pathogen recognition, cytokine production, and initiation of adaptive immunity. These cells exhibit significant plasticity, differentiating into various effector cells, including macrophages and dendritic cells, to adapt to the ever-changing septic environment [13–15]. The functional state of monocytes is a key factor determining the progression and outcomes of sepsis [16]. A deeper understanding of the biology of monocytes in sepsis may reveal new therapeutic strategies to modulate their activity [17]. The bidirectional nature of monocytes in promoting inflammation and resolution and repair emphasizes the need to elucidate the regulatory networks influencing their function [18]. Despite extensive research on various signaling pathways, such as NF-κB and STAT, exploring alternative pathways regulating monocyte responses in sepsis remains a crucial research area [19].

Plac8 is a multifunctional protein involved in cell proliferation, differentiation, and immune regulation. Recently, it has garnered attention due to its potential role in infectious diseases [20, 21]. Preliminary research indicates that Plac8 plays a role in the regulation of immune cell functions; however, the exact mechanism by which it operates in this capacity is not fully comprehended [22, 23]. The involvement of this protein in autophagy, cell cycle control, and apoptosis implies its potential key role in sepsis pathogenesis [24]. Particularly in the context of monocytes, understanding how Plac8 influences their activity by modulating intracellular signaling is crucial [23, 25]. Within the extracellular signal-regulated kinase (ERK) signaling pathway, ERK activation promotes cell survival, proliferation, and differentiation, and is associated with the development of various diseases including infections and autoimmune disorders [26, 27]. In innate immune responses, the ERK pathway plays a crucial role in regulating cytokine production, cell migration, and functional effector differentiation. Therefore, the ERK pathway stands as a potent candidate mechanism for modulating monocyte function and shaping sepsis immune responses [28]. Previous studies have suggested that Plac8 participates in cell fate determination through the ERK pathway [29, 30]. Furthermore, the Plac8/ERK signaling pathway has been confirmed to be involved in regulating cell proliferation [31], yet its role in sepsis remains insufficiently explored. Thus, further research on the interaction between the ERK pathway and Plac8 is crucial for unraveling the complex network of immune cell function in sepsis pathology, providing new insights that could potentially form the basis for future targeted intervention strategies.

This study aims to enhance our understanding of the mechanisms underlying monocyte responses in sepsis, facilitate the translation from basic science to clinical practice, and strengthen our ability to combat this formidable enemy. Ultimately, this research intends to bridge the gap between basic science and clinical practice and enhance our arsenal against sepsis.

## Results

### Characteristics and blood evaluation of patients with septicemia

A total of 28 septicemia patients were included, comprising 15 males and 13 females, with an age range of 18–60 years and a mean age of 38.18 ± 12.97. Additionally, 18 normal controls were recruited, including 10 males and 8 females, with an age range of 28–64 years and a mean age of 45.44 ± 12.34. Statistical analysis revealed no significant differences in age or gender between septicemia patients and normal controls. The sepsis patients had a Sequential Organ Failure Assessment (SOFA) score of 6.54 ± 2.89. Details regarding complications and site of infection in septicemia patients can be found in Table S2.

### Altered phenotype and function of PBMCs in patients with septicemia

**Fig. 1:**
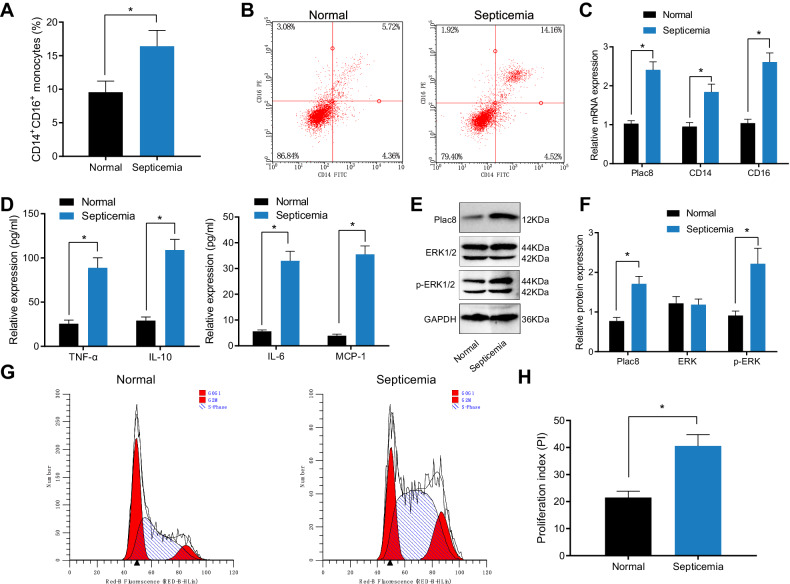
Expression of plac8 and ERK activation in peripheral blood mononuclear cells of septic patients and normal individuals. **A** Flow cytometry analysis of peripheral blood CD14+ and CD16+ cell subsets. **B** Flow cytometry analysis of CD14+ and CD16+ cell subsets. **C** qRT-PCR analysis of gene expression. **D** ELISA analysis of cytokine expression. **E** Western blot analysis of protein expression. **F** Western blot analysis of protein expression. **G** Flow cytometry analysis of cell cycle. **H** Cell proliferation index PI = (S + G2M)/(G0/1 + S + G2M). * indicates *P* < 0.05 compared to the normal group. Data is presented as mean ± standard deviation. Data analysis was performed using an independent samples *t*-test, and the experiment was repeated three times.

Monocytes were isolated from the peripheral blood of septic patients and healthy individuals using Ficoll density gradient centrifugation. Compared to monocytes from healthy individuals, the proportion of CD14+ and CD16+ monocytes in the peripheral blood of septic patients was higher (Fig. 1A, B) (*P* < 0.05). qRT-PCR analysis revealed higher expression levels of the marker genes CD14, CD16, and plac8 (Fig. 1C) (*P* < 0.05). Western blot analysis demonstrated higher expression levels of the plac8 protein (Fig. 1E, F) (*P* < 0.05), with increased phosphorylation of ERK1/2 protein without significant changes in its expression levels (*P* < 0.05). ELISA results indicated higher expression levels of monocyte chemoattractant protein-1 (MCP-1), tumor necrosis factor-alpha (TNF-α), interleukin-6 (IL-6), and anti-inflammatory cytokine IL-10 (Fig. 1D) (*P* < 0.05). Flow cytometry analysis showed a decrease in the proportion of cells in the G0/G1 phase and an increase in the proportion of cells in the S phase, accompanied by an elevated cell proliferation index (PI) with statistical significance (*P* < 0.05) (Fig. 1G, H). No significant differences were observed in the G2 phase of the cells (*P* > 0.05).

### In vitro model of septicemia using LPS-stimulated PBMCs from healthy individuals

To construct an in vitro sepsis model, PBMCs from healthy individuals were stimulated with lipopolysaccharide (LPS). To confirm the model’s success, qRT-PCR analysis revealed higher expression levels of CD14, CD16, and plac8 genes in the LPS-stimulated group compared to the normal group (Fig. S1A, *P* < 0.05). Western blot analysis showed elevated expression of plac8 protein in the LPS-stimulated group compared to the normal group (Fig. S1B, *P* < 0.05), with no significant changes in the protein content of ERK1/2 but an increased phosphorylation level (*P* < 0.05). ELISA results demonstrated increased expression levels of TNF-α, IL-6, IL-10, and MCP-1 in the LPS-stimulated group compared to the normal group (Fig. S1C, *P* < 0.05). Furthermore, using the CCK-8 assay, it was observed that cell proliferation in the LPS-stimulated group was enhanced in comparison to the normal group (Fig. S1D, *P* < 0.05). These results indicate that LPS stimulation induces sepsis-like responses in mononuclear cells and influences their proliferation.

### Plac8 modulation influences survival and proliferation of monocytes in an in vitro model of septicemia

**Fig. 2:**
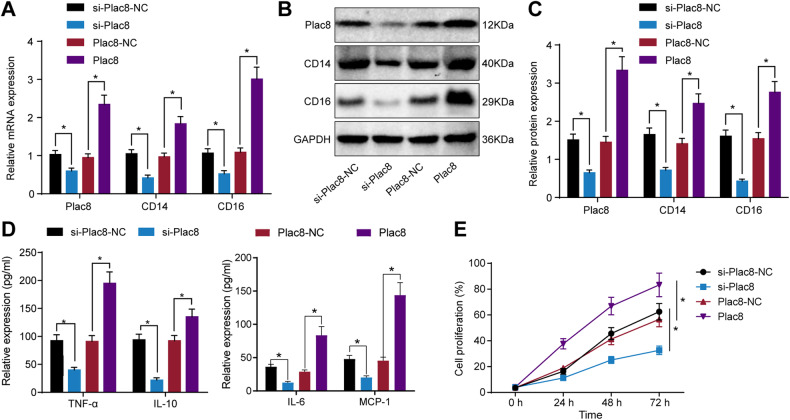
Effects of plac8 on peripheral blood mononuclear cell proliferation in septic patients. **A** qRT-PCR analysis of gene expression. **B** Western blot analysis of protein band. **C** Western blot analysis of protein expression. **D** ELISA analysis of cytokine expression. **E** Cell proliferation measured by CCK-8 assay, cell proliferation (%) = [OD (treatment group) − OD (blank group)] / [OD (control group) − OD (blank group)] × 100%. * indicates *P* < 0.05 compared to si-Plac8-NC or Plac8-NC group. Data is presented as mean ± standard deviation. An independent samples *t*-test was used to compare two groups, and two-way ANOVA was used to compare at different time points. The experiment was repeated three times.

In this study, we established an in vitro sepsis model by stimulating PBMCs from healthy individuals with LPS. The qRT-PCR results (Fig. 2A) showed a significant decrease in the expression levels of plac8, CD14, and CD16 genes in the si-Plac8 group compared to the si-plac8-NC group (*P* < 0.05). In contrast, the plac8 group exhibited a significant increase in the expression levels of plac8, CD14, and CD16 genes compared to the plac8-NC group (*P* < 0.05). Western blot analysis (Fig. 2B, C) revealed a decrease in the protein expression levels of plac8, CD14, and CD16 in the si-Plac8 group compared to the si-plac8-NC group (*P* < 0.05). Conversely, the plac8 group exhibited an increase in the protein expression levels of plac8, CD14, and CD16 compared to the plac8-NC group (*P* < 0.05). ELISA results (Fig. 2D) demonstrated a decrease in the protein expression levels of TNF-α, IL-6, IL-10, and MCP-1 in the si-Plac8 group compared to the si-plac8-NC group (*P* < 0.05). Similarly, the plac8 group exhibited an increase in the protein expression levels of TNF-α, IL-6, IL-10, and MCP-1 compared to the plac8-NC group (*P* < 0.05). Additionally, the CCK-8 assay (Fig. 2E) showed a decrease in cell proliferation of mononuclear cells in the si-Plac8 group compared to the si-plac8-NC group (*P* < 0.05). Conversely, the plac8 group exhibited increased mononuclear cell proliferation compared to the plac8-NC group (*P* < 0.05). These findings indicate that upregulation of plac8 enhances the survival and proliferation of mononuclear cells in sepsis patients, while downregulation of plac8 reduces their survival and proliferation, suggesting that plac8 influences the survival and proliferation of mononuclear cells in sepsis patients.

### Plac8 modulation via ERK pathway affects survival, proliferation, and activation of monocytes in septicemia

**Fig. 3:**
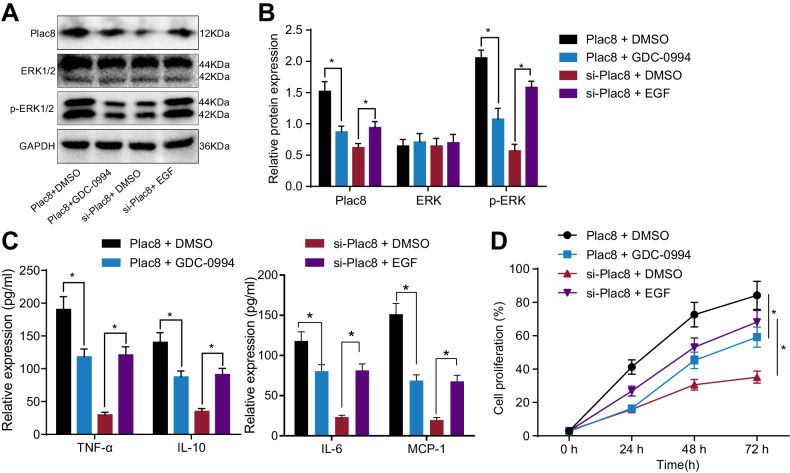
Plac8 affects septic mononuclear cell proliferation by regulating the ERK pathway. **A** Western blot analysis of protein band. **B** Western blot analysis of protein expression. **C** ELISA analysis of cytokine expression. **D** Cell proliferation was measured by CCK-8 assay. * indicates *P* < 0.05 compared to Plac + DMSO or si-Plac + DMSO group. Data is presented as mean ± standard deviation. Independent samples *t*-test was used to compare two groups, and two-way ANOVA was used for comparison at different time points. The experiment was repeated three times.

Western blot analysis revealed that (Fig. 3A, B), compared to the Plac8 + DMSO group, the Plac8 + GDC-0994 group demonstrated a significant decrease in plac8 protein levels (*P* < 0.05). Additionally, the phosphorylation levels of ERK1/2 proteins decreased in the Plac8 + GDC-0994 group, while the protein content remained unchanged. In contrast, in the si-Plac8 + EGF group, plac8 protein expression levels were significantly increased (*P* < 0.05). Similarly, the protein content of ERK1/2 remained unchanged, but the phosphorylation levels increased. ELISA results (Fig. 3C) showed that, compared to the Plac8 + DMSO group, the Plac8 + GDC-0994 group had decreased protein levels of pro-inflammatory factors TNF-α, IL-6, IL-10, and MCP-1 (*P* < 0.05). Conversely, compared to the si-Plac8 + DMSO group, the si-Plac8 + EGF group exhibited increased protein levels of these pro-inflammatory factors (*P* < 0.05). Cell proliferation, measured using the CCK-8 assay (Fig. 3D), showed a significant decrease in monocyte proliferation in the Plac8 + GDC-0994 group when compared to the Plac8 + DMSO group (*P* < 0.05). Conversely, the si-Plac8 + EGF group demonstrated significantly increased monocyte proliferation compared to the si-Plac8 + DMSO group (*P* < 0.05). These findings suggest that Plac8 influences the survival, proliferation, and activation of peripheral blood monocytes in septic patients through the ERK pathway.

### Construction of a mouse model of sepsis

**Fig. 4:**
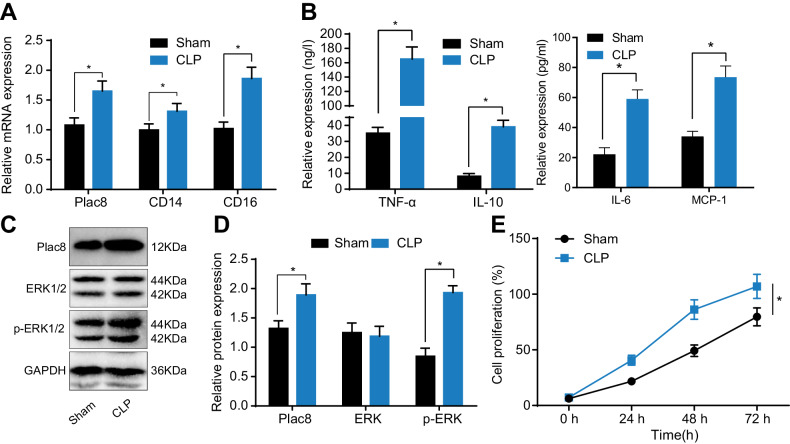
Construction of septic mouse model. **A** qRT-PCR analysis of gene expression. **B** ELISA analysis of relevant factors in mouse serum. **C** Western blot analysis of protein band. **D** Western blot analysis of protein expression. **E** Results of cell proliferation assay using CCK-8 method. * indicates *P* < 0.05 compared to the sham group. Each group consisted of ten mice, and data is presented as mean ± standard deviation. Independent samples *t*-test was used to compare two groups, and two-way ANOVA was used for comparison at different time points.

The success rate of constructing a mouse model of sepsis was 88.89%, with all mice surviving within 6 h. Results obtained from qRT-PCR analysis (Fig. 4A) indicated that, compared to the normal group, the expression levels of plac8, CD14, and CD16 genes were increased in the sepsis model (*P* < 0.05). Western blot analysis (Fig. 4C, D) showed that, compared to the normal group, the protein expression level of plac8 was elevated in the sepsis model (*P* < 0.05), whereas the ERK protein content remained unchanged, but phospho-ERK increased (*P* < 0.05). ELISA results (Fig. 4B) revealed that, compared to the normal group, the protein levels of TNF-α, IL-6, IL-10, and MCP-1 were increased in the sepsis model (*P* < 0.05).

### The influence of Plac8 gene silencing on peripheral blood mononuclear cells in septicemia mice

**Fig. 5:**
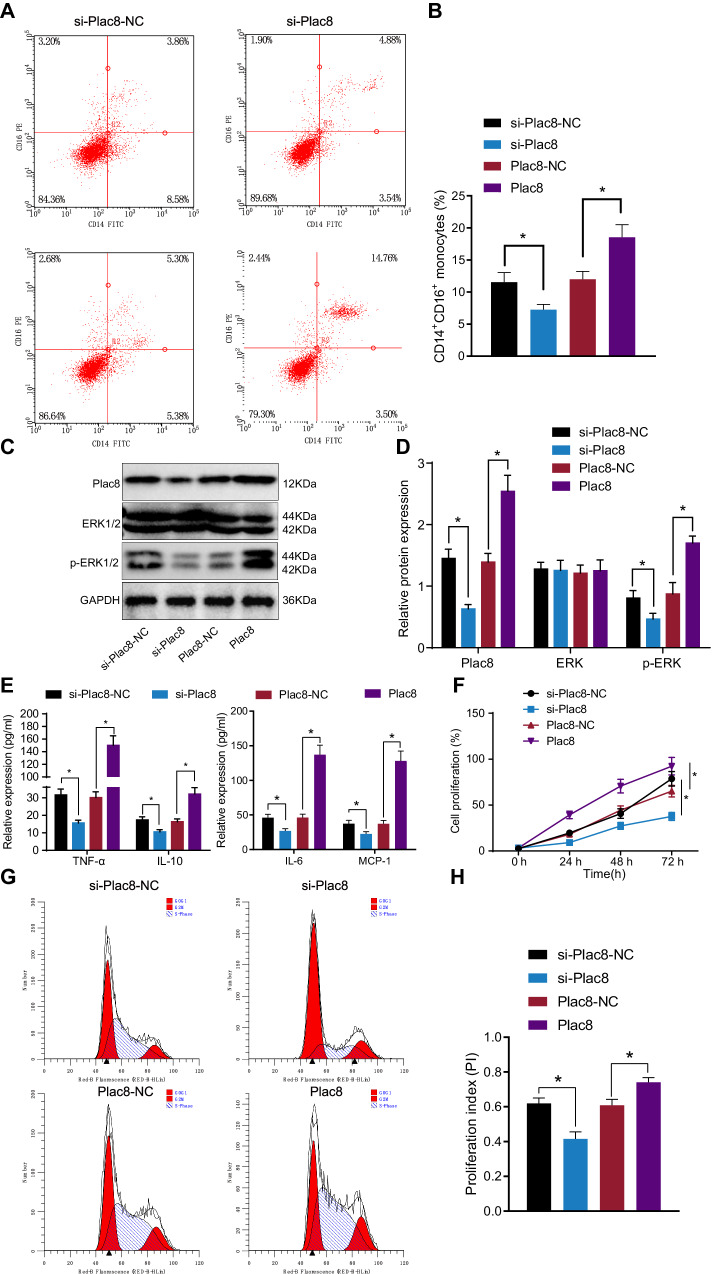
Effects of plac8 on monocyte cell survival and proliferation in the mouse model. **A** Flow cytometry analysis of CD14+ and CD16+ cell subsets. **B** Flow cytometry analysis of peripheral blood CD14+ and CD16+ cell subsets. **C** Western blot analysis of protein band. **D** Western blot analysis of protein expression. **E** ELISA analysis of cytokine expression. **F** Detection of cell proliferation. **G** Flow cytometry analysis of cell cycle. **H** Cell proliferation index PI. * indicates *P* < 0.05 compared to si-Plac8-NC or Plac8-NC group. Data is presented as mean ± standard deviation. Independent samples *t*-test was used to compare two groups, and two-way ANOVA was used for comparison at different time points. The experiment was repeated three times.

In the experimental setup, septic mice were injected with Plac8-siRNA/Plac8 and the corresponding negative control virus, leading to the isolation of mononuclear cells. Flow cytometry analysis demonstrated a decrease in the proportion of CD14+ and CD16+ monocytes in the si-Plac8 group compared to the si-plac8-NC group (*P* < 0.05), whereas an increase was observed in the Plac8 group compared to the Plac8-NC group (*P* < 0.05) (Fig. 5A, B). Western blot analysis revealed a decrease in plac8 protein expression levels in the si-Plac8 group compared to the si-plac8-NC group (*P* < 0.05), accompanied by unchanged levels of ERK protein but decreased phosphorylation (*P* < 0.05). Conversely, an increase in plac8 protein expression levels along with unchanged levels of ERK protein but increased phosphorylation was observed in the Plac8 group compared to the Plac8-NC group (*P* < 0.05) (Fig. 5C, D). ELISA results demonstrated a decrease in the protein expression levels of TNF-α, IL-6, IL-10, and MCP-1 in the si-Plac8 group compared to the si-plac8-NC group (*P* < 0.05), as well as an increase in the Plac8 group compared to the Plac8-NC group (*P* < 0.05) (Fig. 5E). Flow cytometry analysis revealed that the si-Plac8 group exhibited an increase in the proportion of cells in the G0/G1 phase and a decrease in the proportion of cells in the S phase, resulting in a decreased PI with statistical significance (*P* < 0.05) compared to the si-plac8-NC group. On the other hand, the Plac8 group showed a decrease in the proportion of cells in the G0/G1 phase, an increase in the proportion of cells in the S phase, and an elevated PI, all statistically significant (*P* < 0.05) compared to the Plac8-NC group (Fig. 5F–H). These findings strongly suggest the impact of Plac8 on the survival and proliferation of mononuclear cells in the septic mouse model.

## Discussion

The regulation of Plac8 protein expression and its functions in various cell types has been a research focus [32, 33]. Historically, it has been found to play a role in tumor cell proliferation and differentiation, as well as in immune responses. In the inflammatory disease model of sepsis, Plac8 is involved in the activation and apoptosis of inflammatory cells.

**Fig. 6:**
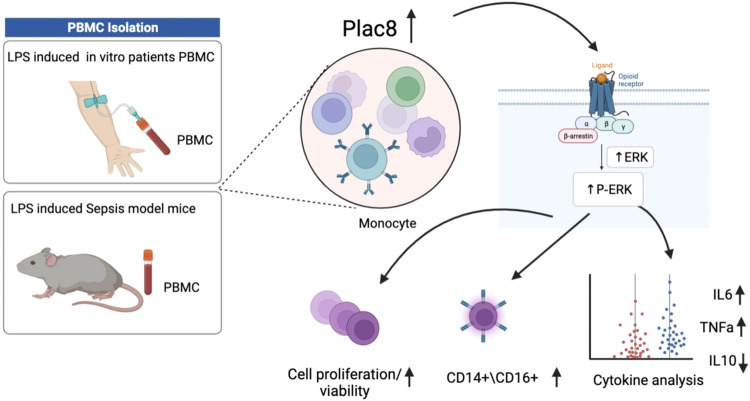
The role and mechanism of Plac8-mediated ERK signaling pathway activation in the proliferation and activation of peripheral blood monocytes in septic patients.

Early research has shown that the ERK pathway plays a central role in cell signaling, particularly in regulating cell proliferation, differentiation, and survival [34–36]. The involvement of the ERK pathway has been widely recognized in monocyte activation and regulation [37, 38]. However, previous studies have not provided a detailed explanation of how Plac8 regulates the ERK pathway [22, 23, 39]. We observed that as the expression of Plac8 increased, the phosphorylation level of ERK protein also increased. It was verified in an in vitro model of LPS-stimulated monocytes and a cecal ligation and puncture (CLP)-induced septic mouse model. It suggests that Plac8 may play a role in sepsis through the signaling pathway mediated by ERK (Fig. 6).

CD14 and CD16, important markers for classifying monocyte subsets, have been extensively studied for their roles in inflammatory responses [40–42]. We observed that the upregulation of Plac8 is not only associated with overall monocyte proliferation activity but also related to changes in the expression of specific phenotypic markers. It may explain the different functions and pathophysiological roles of monocytes in sepsis.

In investigating the regulation of inflammatory responses, we also found that Plac8 may have a bidirectional regulatory function in pro-inflammatory and anti-inflammatory processes. Inflammation is a complex process involving multiple signaling pathways and cell types, and balancing pro-inflammatory and anti-inflammatory signals is crucial [43, 44]. However, further exploration is needed to understand how Plac8 maintains this balance in sepsis, which could have important implications for developing new treatment strategies [24].

In vitro models provide a simplified system for studying cell responses, but they may not fully replicate the complex environment in the human body [45–47]. Similarly, although animal models are indispensable in studying complex disease mechanisms, their results may not always directly extrapolate to humans due to inter-species differences [48, 49]. Future research needs to be conducted in a broader population to validate the role of Plac8 and evaluate its reliability as a biomarker in patients of different ethnicities, ages, and genders [50].

Activation of the ERK pathway significantly influences the proliferation and activation of monocytes by Plac8, providing a novel perspective for understanding the pathophysiology of sepsis. Furthermore, the regulatory role of Plac8 is meaningful for altering the phenotype of monocytes, aiding in uncovering the functional heterogeneity of monocytes in various inflammatory states. These findings expand our scientific understanding of the immune response in sepsis and offer new target molecules for designing therapeutic strategies aimed at modulating monocytes. Building upon the theoretical foundation of this study, clinical applications could explore interventions targeting Plac8 specific to PBMCs to provide effective solutions for precise sepsis treatment.

Limitations of this study primarily include a limited sample size and demographic diversity, potentially impacting the generalizability of the results. While the use of in vitro and animal models facilitates mechanistic exploration, these models do not fully replicate the complex physiological states and immune responses in humans. Moreover, this study has yet to elucidate the molecular mechanisms of Plac8 regulation or consider the interactions of other signaling pathways and their long-term effects. Additionally, uncontrolled variables such as individual differences and lifestyle habits may have influenced the results. Future research efforts should focus on validating the conclusions of this study through increasing sample sizes and conducting multi-center clinical trials. Further investigation into the molecular mechanisms, exploring the specific role of Plac8 in the ERK pathway and its interactions with other signaling pathways to uncover its comprehensive role in immune regulation, will be a key area of future research. Finally, translational medical research should apply these discoveries to the development of therapeutic strategies, ultimately achieving the goal of personalized medicine.

## Materials and methods

### Healthy and sepsis peripheral blood data

Collection of peripheral blood from healthy individuals: fresh peripheral venous blood was collected from 18 healthy voluntary blood donors in our hospital, including 10 males and 8 females, aged 28–64 years and an average age of 45.44 ± 12.34 years.

Collection of peripheral blood from sepsis patients: peripheral blood samples were obtained from 28 sepsis patients admitted to our hospital from January 2020 to December 2020. Among them, 15 were males and 13 were females, with ages ranging from 18 to 60 years and an average age of 38.18 ± 12.97 years. During the collection process, the purpose of the research was explained to the patients or their family members, and informed consent was obtained by having them sign a consent form. Adequate amounts of peripheral blood were drawn from each case.

Inclusion criteria were as follows: (1) patients with a confirmed diagnosis of sepsis; (2) clinical and pathological data available; (3) patients receiving their initial treatment and having not undergone any relevant therapy before. The severity of sepsis was evaluated using the SOFA scoring system. All experiments were conducted in accordance with the principles of the Helsinki Declaration.

### Isolation and culture of blood monocytes

After collecting 1 mL of peripheral blood from patients or healthy individuals and mixing it thoroughly with 1 mL of physiological saline solution, the mixture was slowly added to a test tube containing 2 mL of Ficoll separation solution (F5415, Sigma). The tube was then centrifuged at 1800 rpm for 20 min at room temperature in a horizontal centrifuge (Centrifuge 5804, Eppendorf). Subsequently, the second layer of milky white lymphocytes was carefully transferred to another test tube using a pipette. An equal volume of physiological saline solution was added, and the tube was centrifuged at 1800 rpm for another 20 min using the same centrifuge. This process was repeated three times to isolate monocytes from peripheral blood samples of both healthy individuals and septic patients. Once isolated, the monocytes were suspended in RPMI1640 medium containing 15% FBS (11875119, ThermoFisher) and a 10 μL sample was taken for a trypan blue exclusion test to ensure a cell viability of over 95%. Subsequently, the cells were seeded into 35 mm culture dishes (430165, Corning) and incubated at 37 °C with 5% CO_2_ for 2 h. The culture medium was then aspirated, and the cells were washed five times with preheated 37 °C blank RPMI1640 medium to remove non-adherent cells. A small portion of adherent cells was collected for Wright staining to confirm them as monocytes, comprising 85% of the total cell population. The adherent cells were gently scraped off the culture dish using a cell scraper and transferred to RPMI1640 medium containing 10% FBS (A5669701, ThermoFisher) and 1% P/S (15070063, ThermoFisher), with the cell density adjusted to 1 × 10^6^/ml, and then seeded into 24-well culture plates, 200 μl/well.

### Flow cytometric analysis of MFI in CD14+ CD16+ monocytes

The mean fluorescence intensity (MFI) of CD14+ CD16+ monocytes was measured by flow cytometry. A total of 150 µL of EDTA anticoagulated peripheral whole blood was added to the bottoms of flow tubes, with one tube reserved as a blank control tube without fluorescence antibodies. The remaining tubes were each added with respective antibodies (CD14-PE (ab25390, Abcam, UK) and CD16-FITC (ab124042, Abcam, UK), 10 µL each), and then gently mixed by shaking and incubated at room temperature in the dark. Red blood cell lysis buffer was added to each tube, followed by cell washing. Each tube was then fixed with paraformaldehyde, and the MFI of CD14+ CD16+ was measured using a flow cytometer. Isotype controls were used concurrently to ensure antibody specificity.

### LPS stimulation in an in vitro model

To establish an in vitro model of sepsis, PBMCs from healthy individuals were isolated and stimulated with 8 μg/ml of LPS from Sigma-Aldrich (Merck KGaA, Darmstadt, Germany) for 12 h [51].

### Plasmid transfection

The steps for plasmid transfection were conducted as follows: DNA oligonucleotides were chemically synthesized by Shanghai Jikai Gene Chemistry Technology Co., Ltd. The synthesized DNA was then inserted into a plasmid containing the reporter gene cGFP and the neomycin resistance gene. The day before transfection, the culture medium was replaced with an antibiotic-free medium supplemented with 10% fetal bovine serum. When the cell density reached 70%–80%, transfection was performed according to the instructions provided with Lipofectamine 2000 (Invitrogen, USA, Cat. No. 11668019). The transfected cells were incubated for 4–6 h before replacing the medium with a serum-containing medium. Successful transfection was confirmed by the expression of green fluorescent protein within the cells. After 48 h of transfection, cells were observed using an inverted fluorescence microscope (CFM-500E/CFM-500Z, Shanghai Changfang Optical Instrument Co., Ltd., Shanghai, China) to assess transfection efficiency, and cells were collected for subsequent experiments. The experimental groups for cell transfection were as follows: (1) Plac8-NC group: transfected with the plasmid carrying a nonspecific sequence for Plac8; (2) Plac8 group: transfected with the plasmid overexpressing Plac8; (3) si-Plac8-NC group: transfected with the Plac8 siRNA nonspecific sequence; (4) si-Plac8 group: transfected with the Plac8 siRNA; (5) Plac8 + DMSO group: transfected with the plasmid overexpressing Plac8 and treated with the ERK pathway inhibitor solvent DMSO; (6) Plac8 + GDC-0994 group: transfected with the plasmid overexpressing Plac8 and treated with the ERK pathway inhibitor GDC-0994; (7) si-Plac8 + DMSO group: transfected with Plac8 siRNA and treated with the ERK pathway activator solvent DMSO; (8) si-Plac8 + EGF group: transfected with Plac8 siRNA and treated with the ERK pathway activator EGF ((H)Y-15947, MCE for 24 h; EGF treatment at 200 pg/mL; (H)Y-P7109, MCE) for cells.

### Construction of sepsis mouse model

A total of 80 C57BL/6 laboratory mice were randomly chosen for the study. Ten mice were assigned to the sham operation group, while the remaining 70 mice were used to construct the CLP sepsis mouse model, with 10 mice in each group. The modeling process was conducted in a room at an ambient temperature of 22 °C. For the CLP model group, male C57BL/6 mice were selected and fasted for 8 h without water access. Anesthesia was induced by intraperitoneal injection of the prepared anesthetic agent. The mice were placed supine and fixed on an operating table. The abdominal area was disinfected with 75% ethanol. A midline incision of 2–3 cm in length was made below the xiphoid process along the linea alba to expose the abdominal area. Next, the cecum and the junction of the small and large intestines were located. After the cecum was identified, a circumferential ligation was performed at the distal part of the cecum using 3-0 silk thread, with the ligation width being two-thirds of the width of the ligation site. Then, the intestine was pierced twice with an 8-gauge needle, and the two needle holes were ~3 mm apart to extrude an appropriate amount of intestinal contents. The intestinal contents and cecum were replaced in situ, and the abdominal area was closed layer by layer, with the ligation site being sutured separately. Immediately after the surgery, a subcutaneous injection of 0.9% saline solution at a dose of 5 mL/100 g of body weight was administered to replenish fluid loss during the surgery. The sham operation group was subjected to exposure of intestinal contents without intestinal ligation or puncture.

After successfully constructing the CLP sepsis mouse model, 10 model mice were randomly selected as the CLP control group. Subsequently, an additional 60 mice were randomly divided into four groups, with 15 mice in each group. During the breeding process, each group of mice was injected with their respective lentiviral vectors (1 × 10^9^ pfu/100 μL) via tail injection twice a week for 6 weeks. The mice in the four groups were injected with the following: si-Plac8-NC group (mice injected with si-Plac8-negative control lentiviral vector via tail vein), si-Plac8 group (mice injected with si-Plac8 lentiviral vector via tail vein), Plac8-NC group (mice injected with Plac8-negative control lentiviral vector via tail vein), Plac8 group (mice injected with Plac8 lentiviral vector via tail vein). After the last injection, blood samples were collected from the mice and stored for subsequent experiments 24 h post-injection. Sibiono GeneTech Co., Ltd. provided all lentiviral vectors.

### ELISA

Blood was collected from the abdominal aorta of experimental mice. After 30 min of static incubation, the blood was centrifuged at 3500 rpm for 10 min. ELISA testing was performed to measure the serum levels of pro-inflammatory cytokines TNF-α, IL-6, anti-inflammatory cytokine IL-10, and monocyte chemoattractant protein-1 (MCP-1), following the instructions provided in the BMS623/2 kit (Bender, USA). The known antigens were diluted to a concentration of 1–10 μg/mL and added to each well at a volume of 0.1 mL, which was then kept at 4 °C overnight. The plates were washed three times the next day. Subsequently, a certain volume of the diluted supernatant was added to the coated wells and incubated at 37 °C for 1 h, followed by washing.

Meanwhile, blank, negative, and positive control wells were set up in the reaction wells, and 0.1 mL of freshly diluted enzyme-linked secondary antibody (Abcam) was added. The plates were then incubated at 37 °C for 35–60 min and washed. Finally, the plates were washed again with ddH2O provided by Beijing Dingguo Changsheng Biotechnology Co., Ltd. (Batch No. PER018-1). A total of 0.1 mL of TMB substrate solution (Batch No. EL0001 provided by Huzhou Yingchuang Biotechnology Co., Ltd.) was added to each reaction well and incubated at 37 °C for 10–30 min. The reaction was stopped by adding 50 μL of stop solution, and the absorbance (OD value) of each well was measured at a wavelength of 450 nm within 20 min.

### qRT-PCR

The total RNA of each group of cells was extracted using the one-step Trizol method (15596026, Invitrogen, Car, Cal, USA). The purity and concentration of the RNA were assessed using a UV spectrophotometer (DU640; Backman, USA), and a purity ratio of A260/A280 between 1.8 and 2.0 was considered high. The RNA was then reverse transcribed into cDNA using the PrimeScript RT reagent Kit (RR047A, Takara, Japan) according to the manufacturer’s instructions. The reaction conditions were as follows: 37 °C for 15 min, 85 °C for 5 s, with a total volume of 20 μL. Subsequently, PCR reactions were conducted using the SYBR Premix EX Taq kit (RR420A, Takara) and a real-time fluorescence quantitative PCR instrument (ABI 7500, ABI; Foster City, CA, USA). The reaction mixture contained SYBR Mix 9 μL, upstream primer 0.5 μL, downstream primer 0.5 μL, cDNA 2 μL, and RNase Free dH2O 8 μL. The reaction conditions were as follows: 95 °C for 10 min, followed by 40 cycles of 95 °C for 15 s and 60 °C for 1 min. Each well was set up with 3 replicates. The primers were synthesized by Shanghai Jikai Gene Chemical Technology Co., Ltd. (primer sequences are listed in Table S1). The Ct values of each well were recorded, and the relative expression level of the target gene was calculated using the 2^−ΔΔCt^ method with GAPDH as the internal reference [52]. ΔΔCt = (average Ct value of the target gene in the experimental group − average Ct value of the housekeeping gene in the experimental group) − (average Ct value of the target gene in the control group − average Ct value of the housekeeping gene in the control group).

### Western blot

The cells were cultured until 80% confluency and then lysed on ice for 5 min using RIPA lysis buffer (P0013B, BiyunTian). The lysates were centrifuged at 14,000 rpm at 4 °C, and the supernatant was collected. The protein concentration of the samples was determined using the BCA method (23225, Pierce, USA). Electrophoresis and transfer were performed using 4% stacking gel and 10% resolving gel. After blocking with 0.5% BSA, the membranes were incubated overnight with primary antibodies: rabbit anti-CD14 (ab182032, 1:1000, Abcam, UK), CD16 (Cat No. 16559-1-AP, 1:1000, Proteintech), plac8 (Cat No. 12284-1-AP, 1:200, Proteintech), ERK1/2 (ab17942, 1:1000, Abcam, UK), p-ERK1/2 (ab223500, 1:1000, Abcam, UK), and GAPDH (ab8245, 1:1000, Abcam, UK). After washing, the membranes were incubated with HRP-conjugated goat anti-rabbit IgG (1:2000, ab6721, Abcam, UK) at room temperature for 2 h, followed by visualization using the ECL method (32209, Invitrogen, USA). Images were captured using the Bio-Rad imaging system and analyzed using Image Lab. The experiments above were repeated three times. All the Original WB gels are available at Supplementary files.

### Flow cytometry for cell cycle detection

First, cells were transfected for 48 h and in the logarithmic growth phase, they were washed twice with PBS solution, and the supernatant was discarded. Then, 1 mL of pre-chilled 70% ethanol was added, and cells were fixed overnight at 4 °C. Subsequently, cells were washed twice with PBS solution and suspended in 500 μL of PBS. Propidium iodide (88378, Sigma, USA) and RNase A (GE101-01, Full Shine Biological Co., Ltd., Beijing, China) were added to the cell suspension to reach a final concentration of 50 μg/mL and incubated at 37 °C in a water bath for 30 min. Finally, flow cytometry with red fluorescence detection at an excitation wavelength of 488 nm was used to determine the cell cycle. The PI was calculated using the formula PI = (S + G2M) / (G0/1 + S + G2M).

### CCK-8

The cells were collected and digested using 0.25% trypsin. After counting, they were seeded into a 96-well plate, with a density of 1 × 10^4^ cells/well and 3 replicate wells per group. Each well was added with 10 µL of CCK-8 reagent (40203ES60, Shanghai Yisheng Biotechnology Co., Ltd., China). Subsequently, the absorbance values at 450 nm were measured using an automated microplate reader (MK3, Thermo, USA) at 0, 24, 48, and 72 h to determine cell viability. Cell viability was calculated using the formula (%) = [(OD treatment group) − (OD blank)] / [(OD control group) − (OD blank)] × 100%. The experiment was replicated three times.

### Statistical analysis

All data were processed using the GraphPad prism statistical software. Following normality assessment (Shapiro–Wilk test) and homogeneity of variances test (Levene’s test), quantitative data conforming to a normal distribution were presented as mean ± standard deviation. Differences between the two groups were compared using the unpaired Student’s *t*-test, while one-way analysis of variance (ANOVA) was utilized for comparisons among multiple groups. Additionally, Tukey’s correction and other multiple comparison methods were employed to determine specific intergroup differences’ significance. In the case of non-normally distributed or heteroscedastic data, significant differences were evaluated using the Mann–Whitney *U* test or the Kruskal–Wallis *H* test. Cell viability at different time points was analyzed using two-way analysis of variance. A *p* value < 0.05 indicated statistically significant differences. All experiments were conducted independently at least three times.

### Supplementary information

Supplementary files

## Data Availability

The data that support the findings of this study are available on request from the corresponding author.
