# Supplementary material for: Plac8-ERK pathway modulation of monocyte function in sepsis
Source: Cell Death Discov. 2024 Jul 3;10:308. doi: 10.1038/s41420-024-02012-4 (PMC11222481; doi:10.1038/s41420-024-02012-4)
Supplement: Supplementary file 1 — Supplementary files [file 41420_2024_2012_MOESM1_ESM.docx]

**
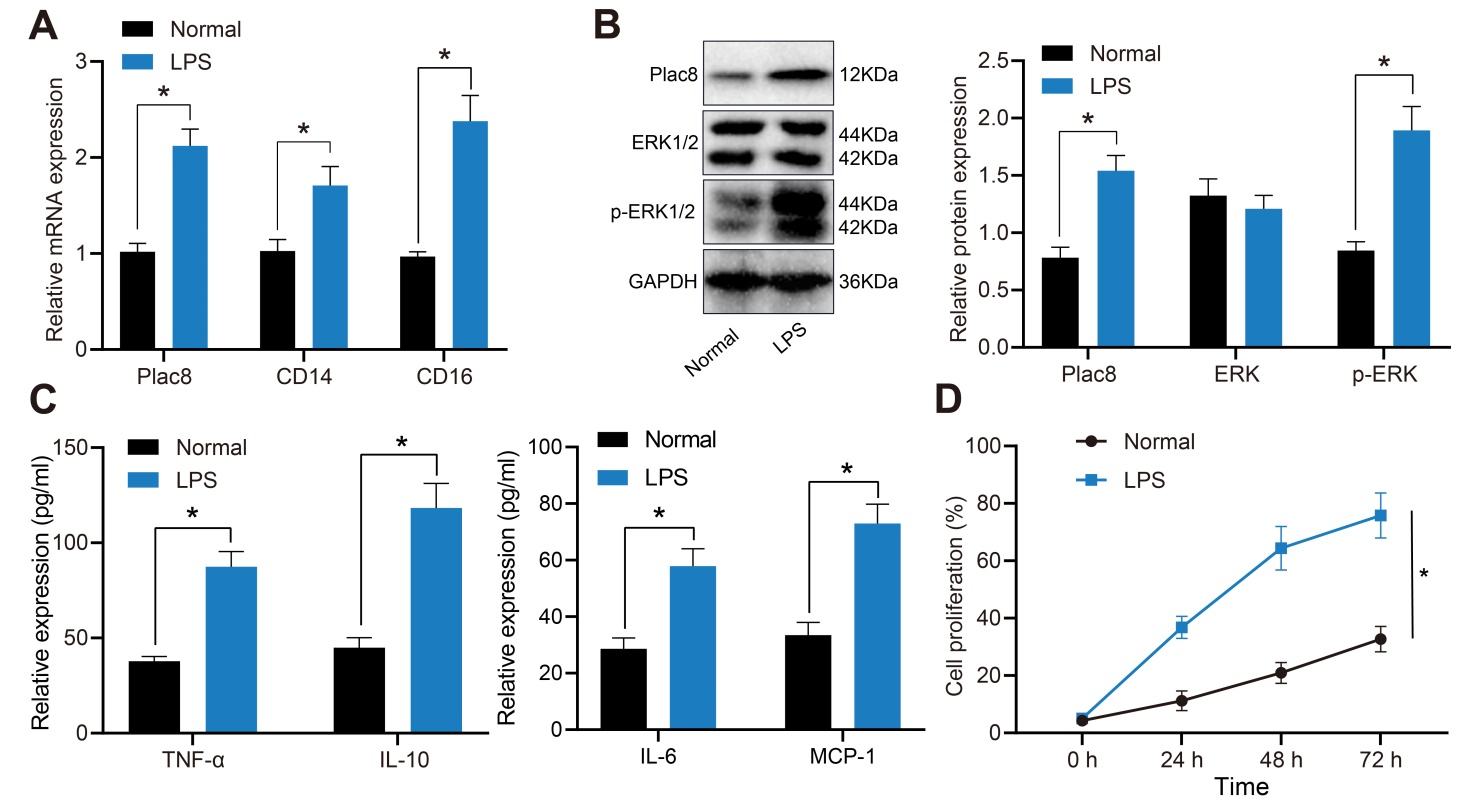
**

**Fig. S1. LPS-Stimulated in vitro Sepsis Model in Monocytes**

Note: (A) qRT-PCR analysis of gene expression. (B) Western blot analysis of protein expression. (C) ELISA measurement of cytokine expression. (D) Cell proliferation assessed by CCK-8 assay, where cell proliferation (%)=[OD (treatment group) - OD (blank group) / OD (control group) - OD (blank group)]×100%. * indicates P<0.05 compared to the Normal group. Data are presented as mean ± standard deviation. Independent sample t-tests were used for intergroup comparisons, while two-way ANOVA was employed for different time points. The experiment was repeated three times.

**Table S1** Primer sequences for reverse transcription quantitative polymerase chain reaction

| Gene | Primer sequence |
| --- | --- |
| CD14+ | F: ACTTCTCAGATCCGAAGCCAG |
|  | R: CCGCCGTACAATTCCACAT |
| CD16+ | F: AATGCACACTCTGGAAGCCAA |
|  | R: CACTCTGCCTGTCTGCAAAAG |
| Plac8 | F: CACCAACAGTTATCGTGACTCA |
|  | R: CCACACAGACAACACTCATTCA |
| GAPDH | F: AGGTCGGTGTGAACGGATTTG |
|  | R: GGGGTCGTTGATGGCAACA |

Note: F, forward; R, reverse; Plac8, placenta-specific 8; GAPDH, glyceraldehyde-3-phosphate dehydrogenase; CD, cluster of differentiation.

**Table S2** Baseline characteristics of study subjects

| **Characteristics** | Sepsis patients | Healthy donors | *p* |
| --- | --- | --- | --- |
|  | N = 28 | N = 18 |  |
| Age (year) | 38.18 ± 12.97 | 45.44 ± 12.34 | 0.066 |
| Gender (male) N (%) | 15 (53.27%) | 10 (55.55%) | 0.895 |
| SOFA score | 6.54 ± 2.89 |  |  |
| Complication N (%) |  |  |  |
| Hypertension | 7 (25.00%) |  |  |
| Diabetes | 13 (46.43%) |  |  |
| Heart disease | 7 (25.00%) |  |  |
| Chronic kidney disease | 1 (3.57%) |  |  |
| Infection site N (%) |  |  |  |
| Lung | 8 (28.57%) |  |  |
| Abdomen | 2 (7.14%) |  |  |
| Bone or soft tissue | 4 (14.29%) |  |  |
| Wound infection | 2 (7.14%) |  |  |
| Urogenital | 11 (39.29%) |  |  |
| Others | 1 (3.57%) |  |  |

Notes: SOFA, sequential organ failure assessment; Continuous variables are represented by mean and standard deviation, and classified variables are represented as number and percentage. The difference analysis of age between sepsis patients and controls was tested by *t* test, and the difference analysis of gender between sepsis patients and controls was tested by chi square test. *p* < 0.05 indicated that the difference was statistically significant.

Original WB gels


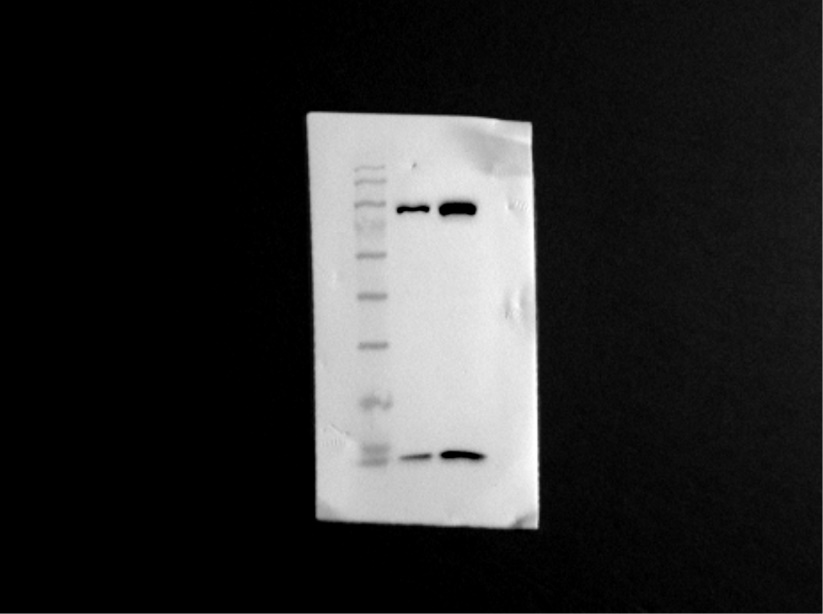


Figure 1E-1


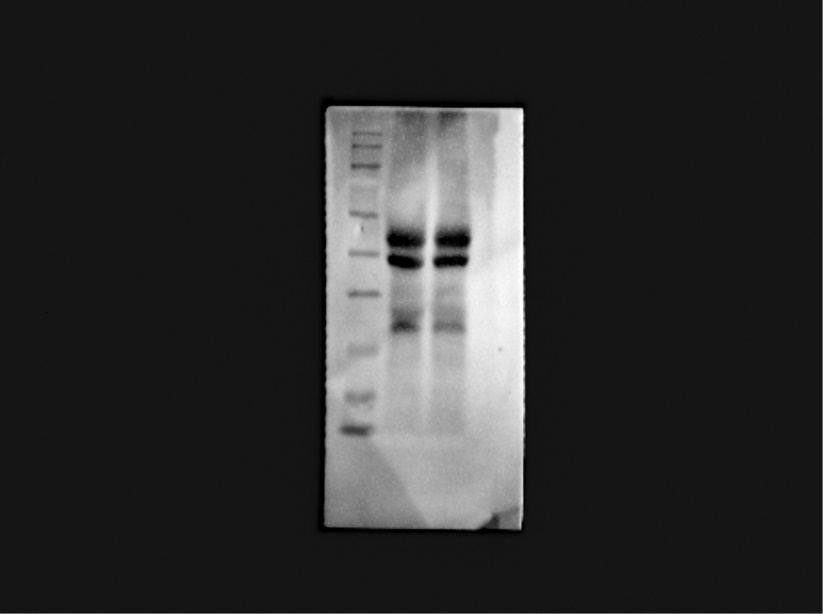


Figure 1E-2


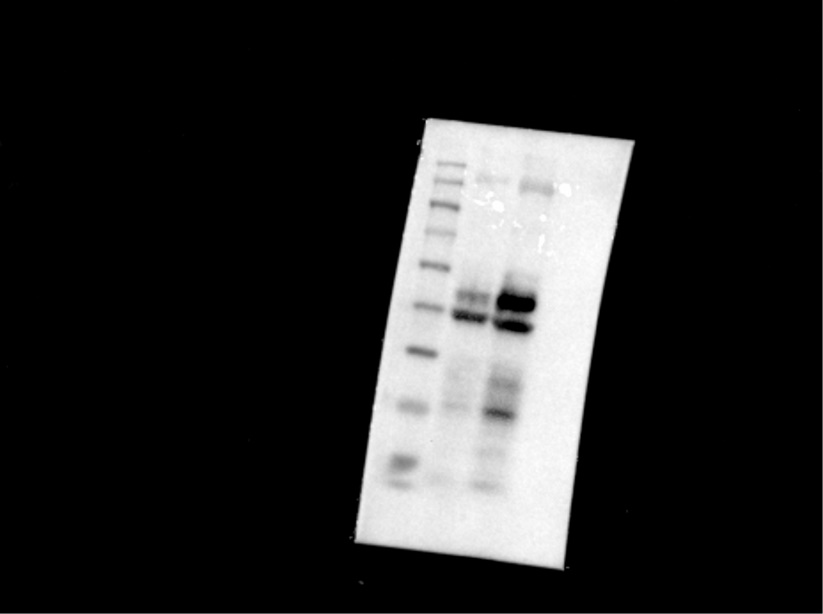


Figure 1E-3


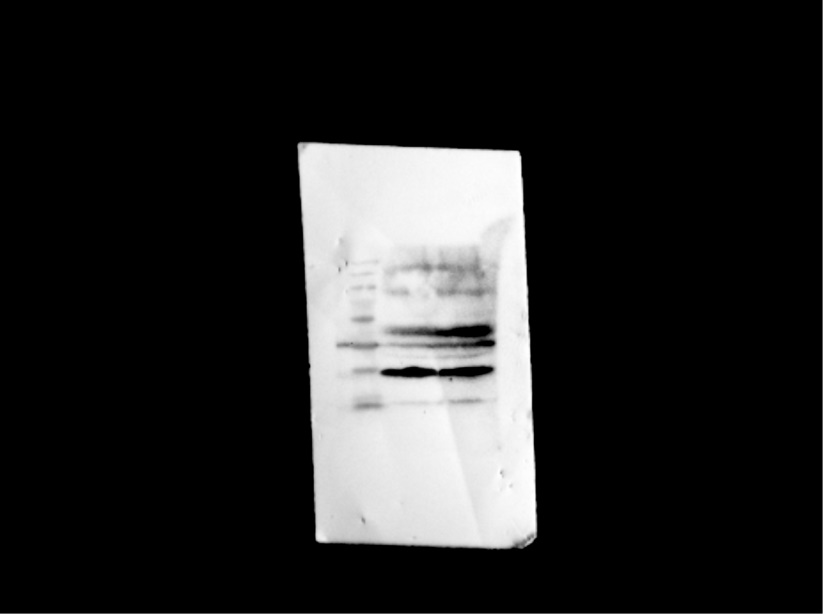


Figure 1E-4


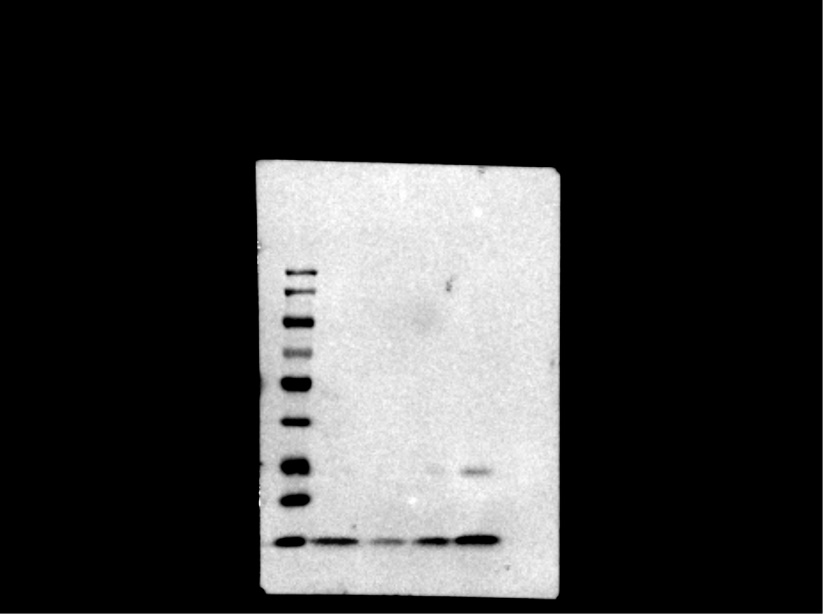


Figure 2B-1


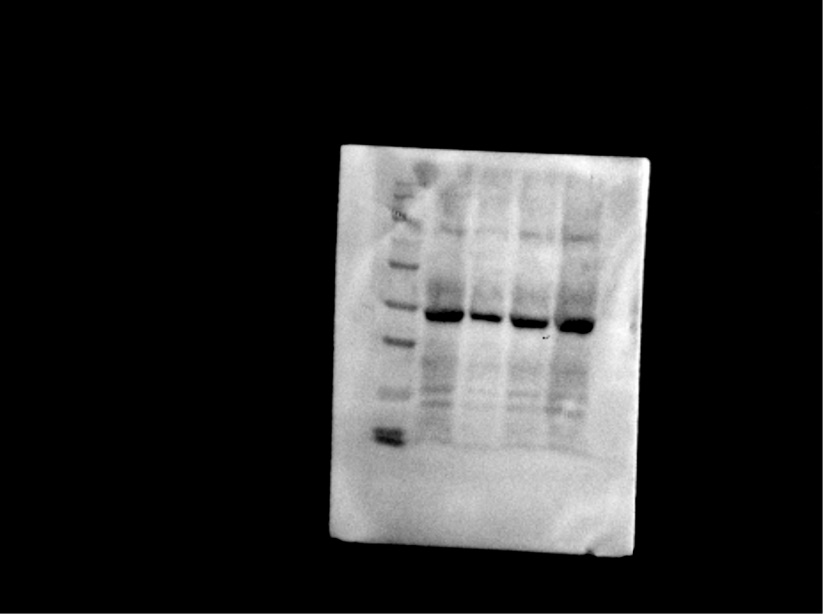


Figure 2B-2


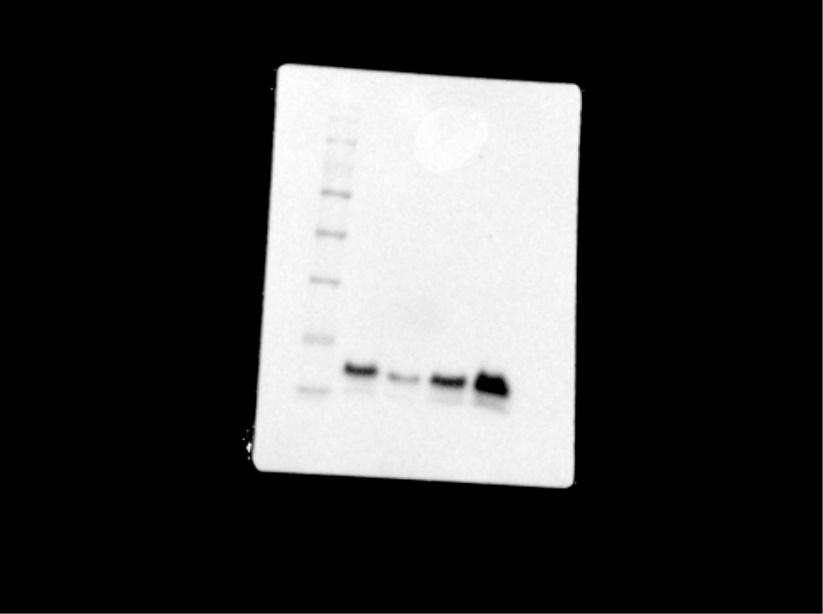


Figure 2B-3


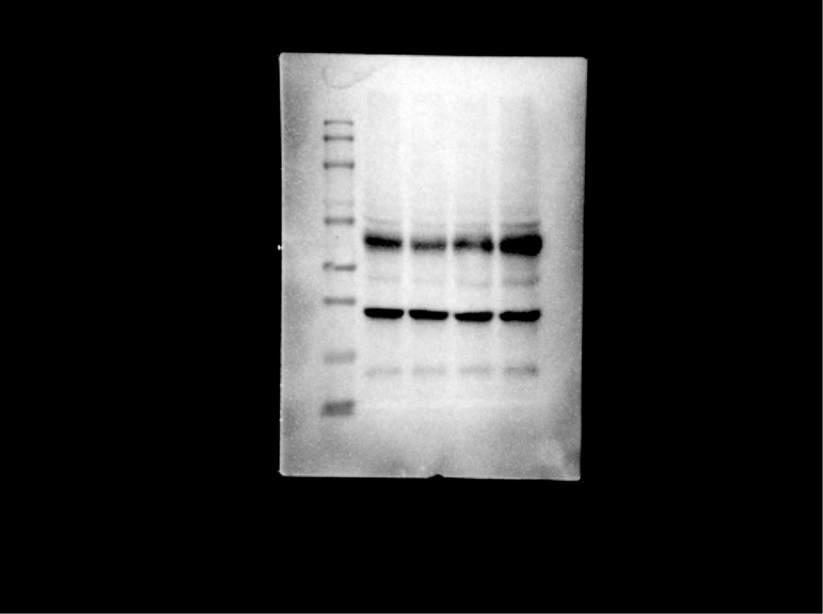


Figure 2B-4


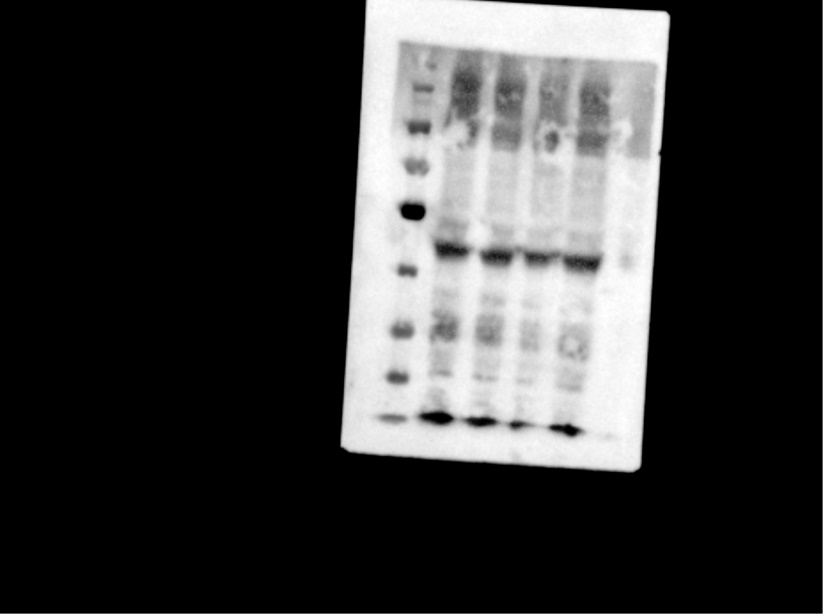


Figure 3A-1


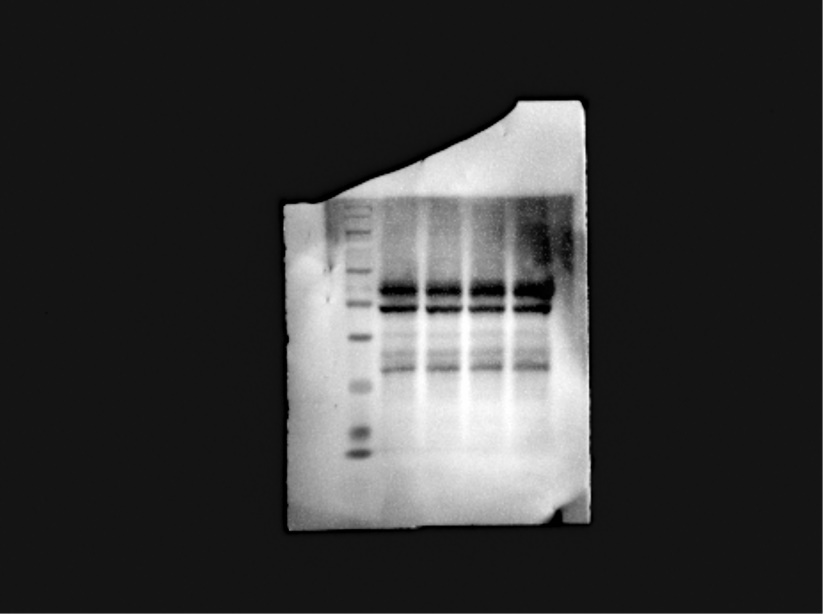


Figure 3A-2


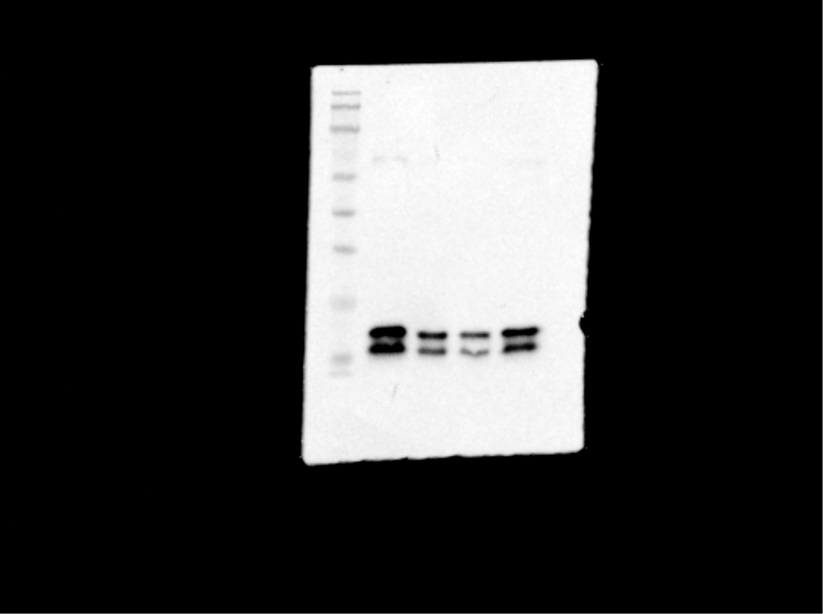


Figure 3A-3


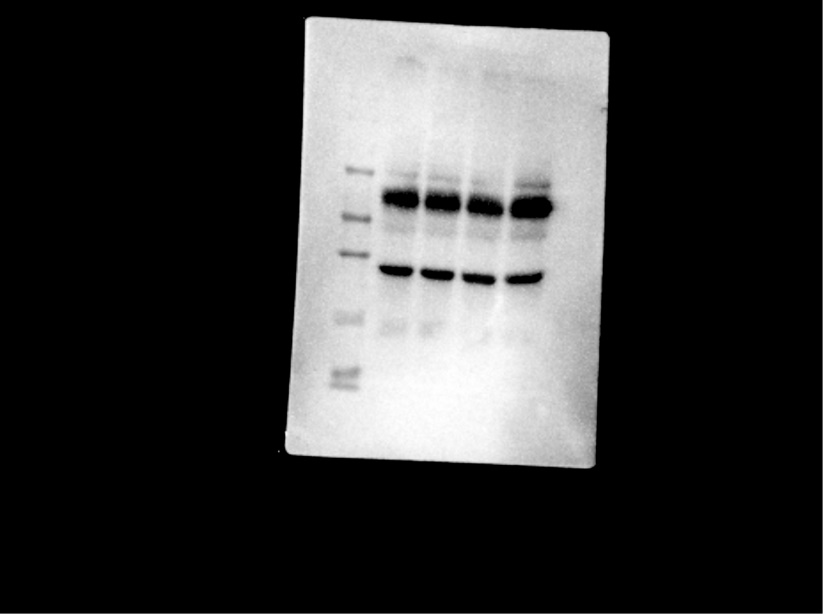


Figure 3A-4

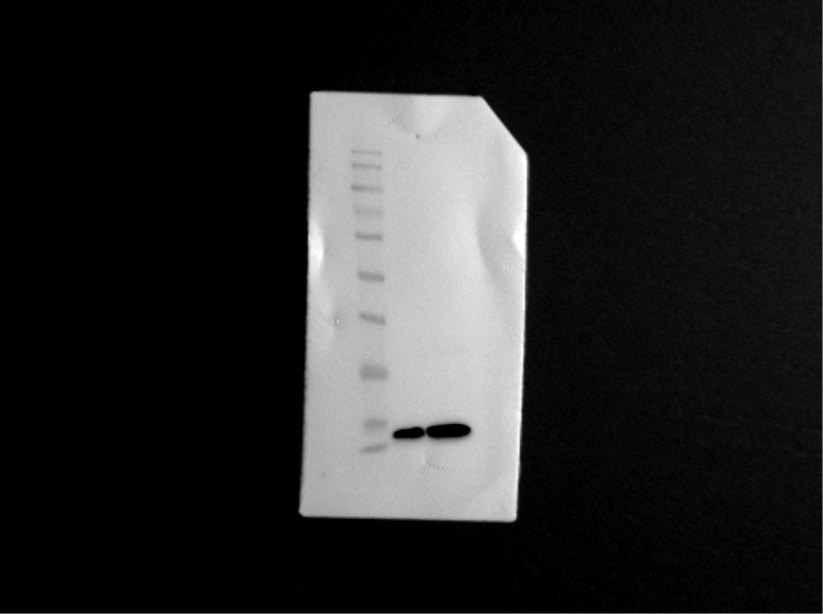


Figure 4C-1


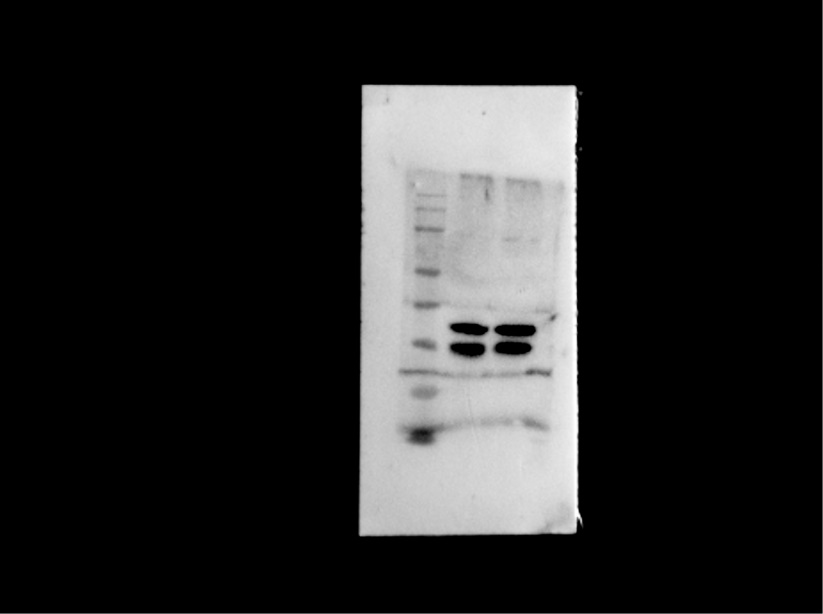


Figure 4C-2


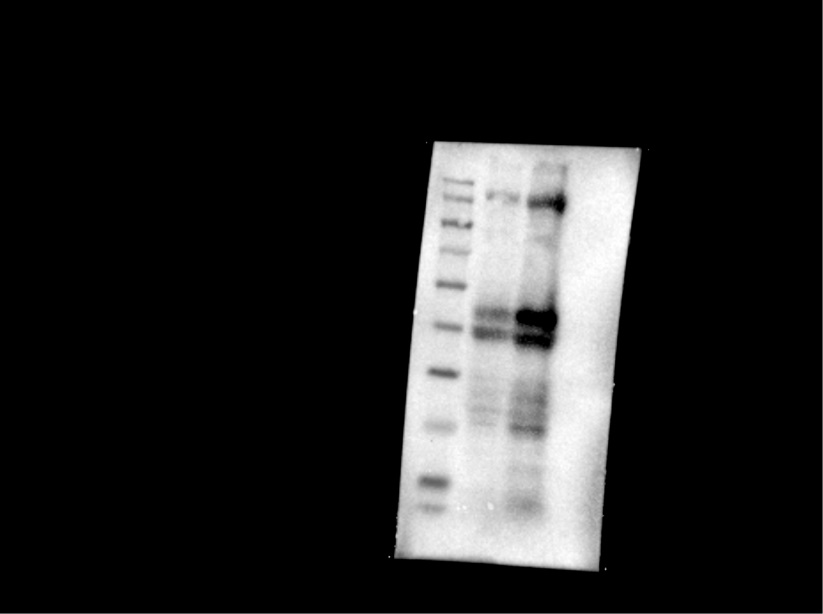


Figure 4C-3


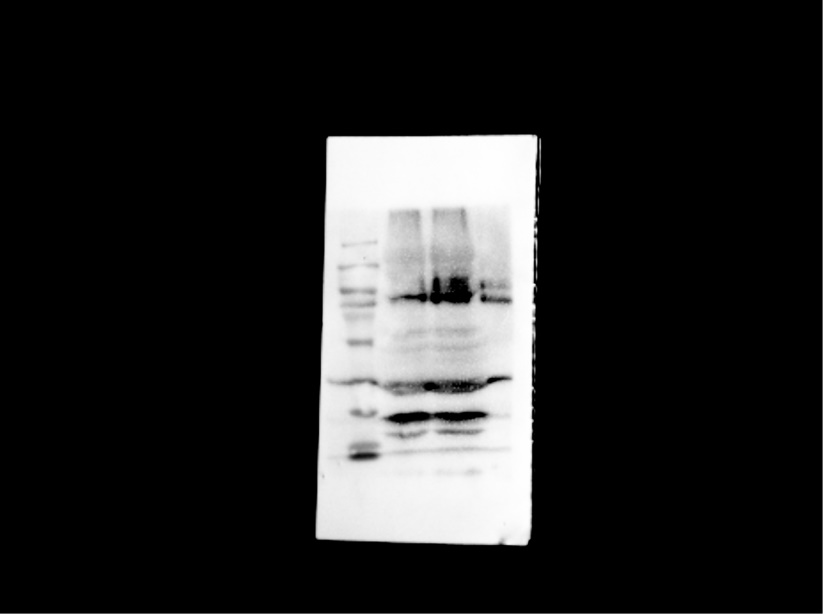


Figure 4C-4


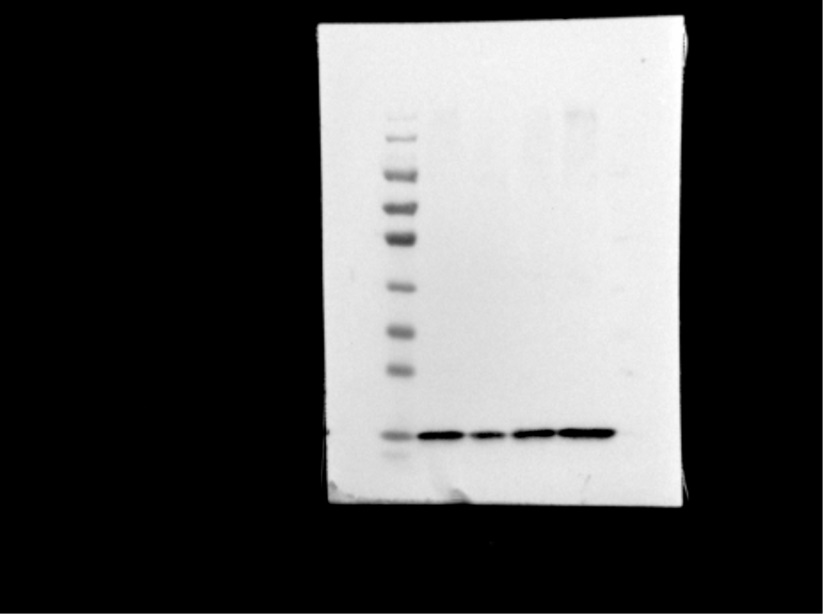


Figure 5C-1


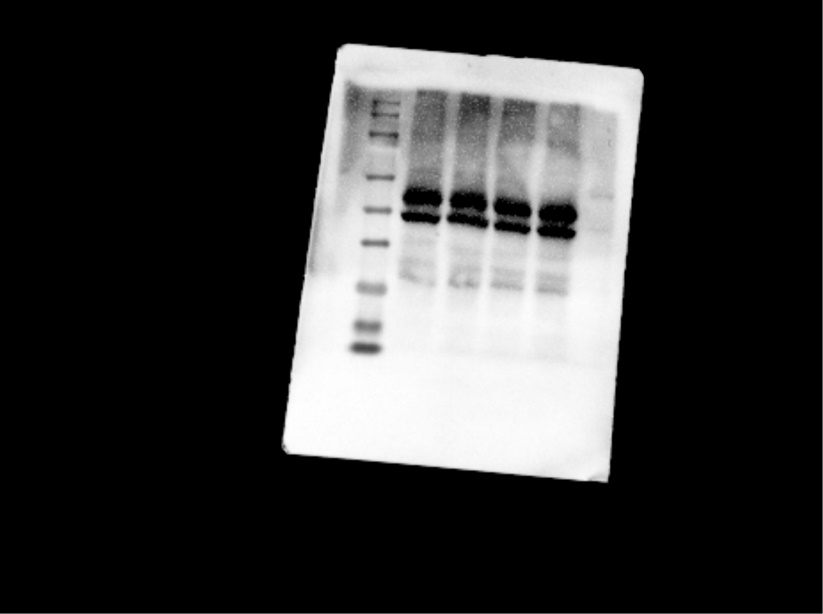


Figure 5C-2


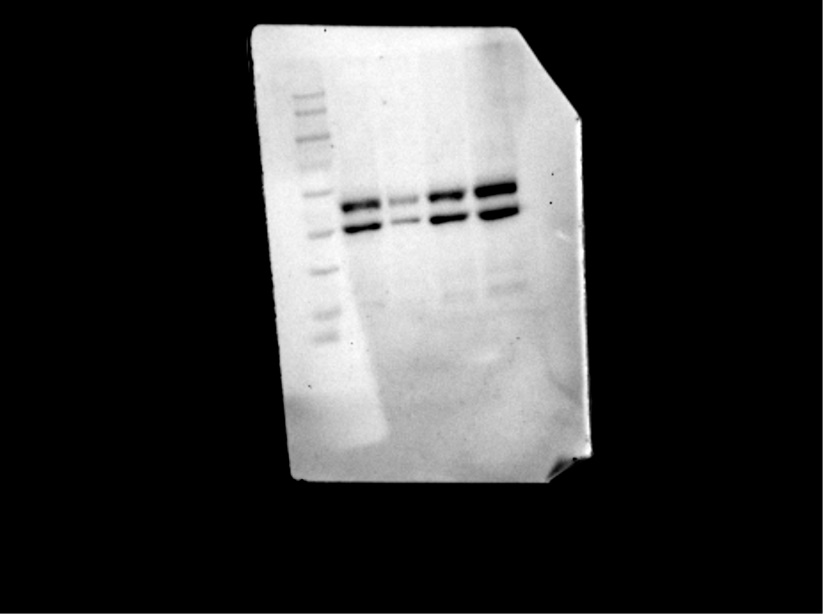


Figure 5C-3


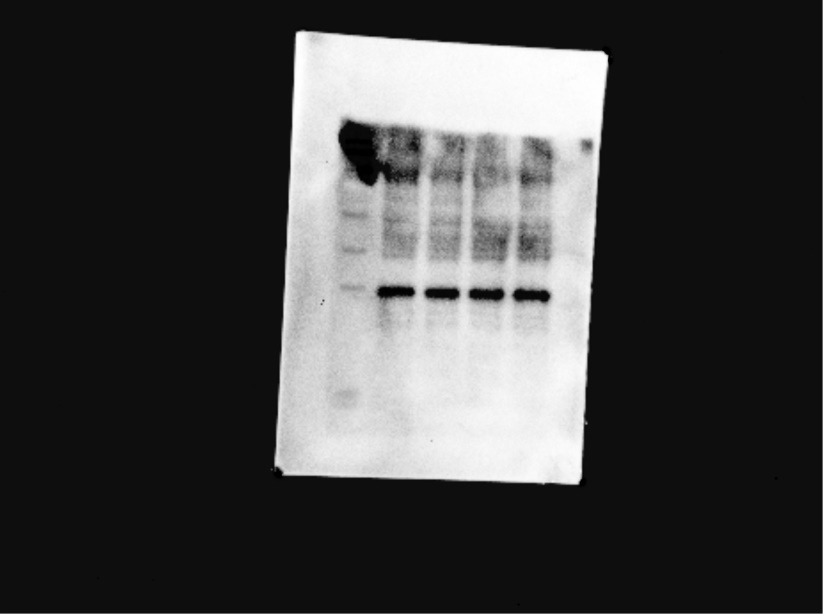


Figure 5C-4


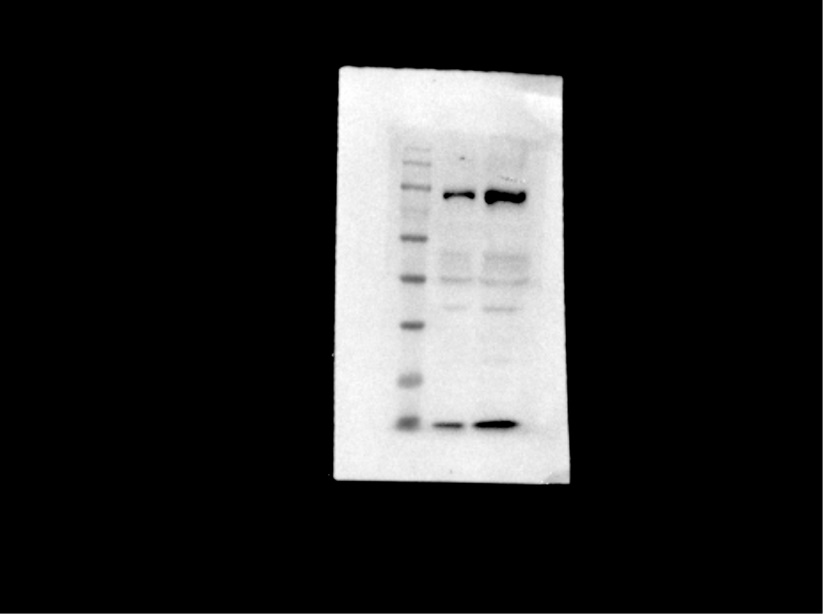


Figure S1B-1


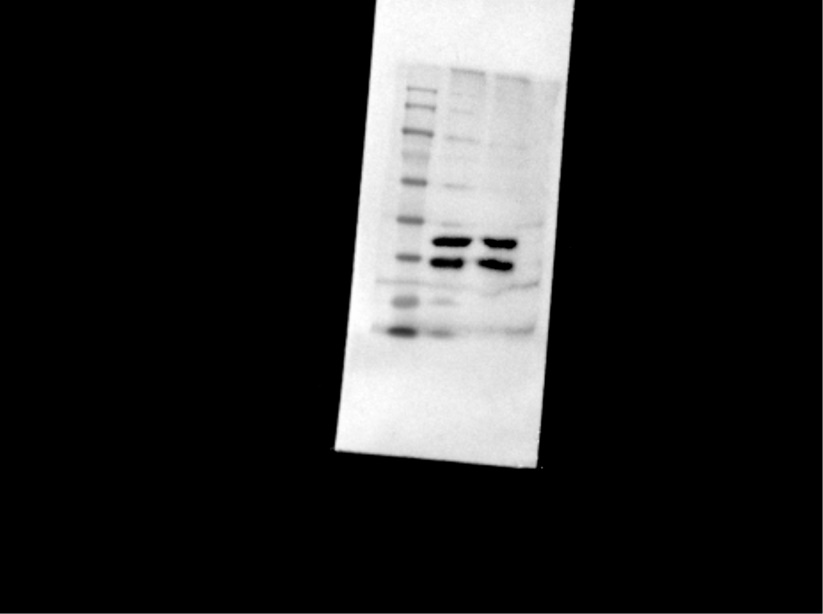


Figure S1B-2


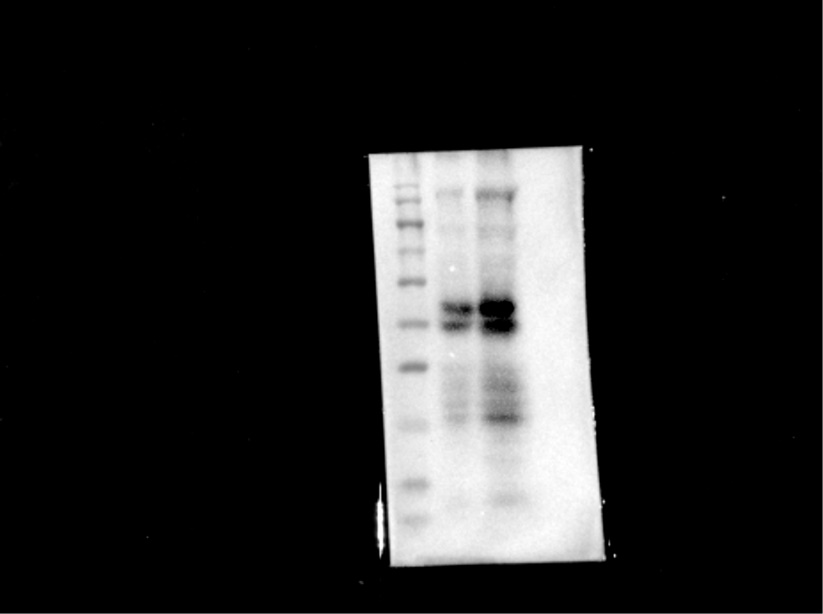


Figure S1B-3


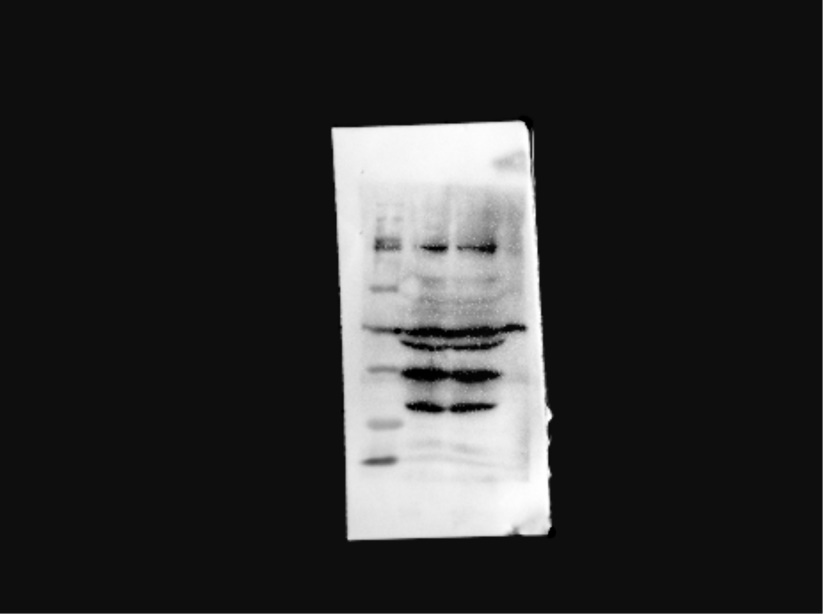


Figure S1B-4
